# Outcome of management of brain abscess in children

**DOI:** 10.12669/pjms.36.3.1087

**Published:** 2020

**Authors:** Ikram Ullah Khan, Abdul Latif, Muhammad Ashraf, Muhammad Kashif Chishti, Sadia Sadiq

**Affiliations:** 1 Ikram Ullah Khan, Assistant Professor, Department of Paediatric Surgery, The Children’s Hospital & The Institute of Child Health, Multan, Pakistan; 2 Abdul Latif, Associate Professor, Department of Paediatric Surgery, Nishtar Medical University, Multan, Pakistan; 3 Muhammad Ashraf, Associate Professor, Department of Paediatric Neurosurgery, The Children’s Hospital & The Institute of Child Health, Multan, Pakistan; 4 Muhammad Kashif Chishti Professor of Paeds: Surgery, The Children’s Hospital & The Institute of Child Health, Multan, Pakistan; 5 Sadia Sadiq, Department of Radiology, The Children’s Hospital & The Institute of Child Health, Multan, Pakistan

**Keywords:** Brain abscess, Children, Craniotomy

## Abstract

**Objective:**

To find out the outcome of management of brain abscess in children.

**Methods:**

This is prospective observational study conducted in the Department of Paediatric Neurosurgery at Children’s Hospital and Institute of Child Health, Multan from July 2014 to June 2017. Children up to the age of 14 years suffering from brain abscess were admitted. After taking clinical history, general and systemic physical examination and necessary investigations, abscess was evacuated and abscess wall excised after performing craniotomy. Data was collected on a predesigned performa. Results were analyzed and compared with national and international literature through statistical package for social sciences (SPSS-20).

**Results:**

Twenty five patients up to 14 years of age were included. Seventeen (68%) were male and eight (32%) female. Fever and vomiting were present in all 25 (100%) patients. Paranasal sinusitis was predisposing causative factor in 9(36%) followed by otitis media in 7 (28%). Abscess was present in frontal lobe in 9 (36%), temporoparietal region in 8 (32%), posterior fossa in 5 (20%) and multiple abscesses in 3 (12%). Craniotomy was performed, pus evacuated and abscess wall excised in all 25 (100%) patients. Three (12%) patients expired.

**Conclusion:**

Incidence of brain abscess can be decreased by treatment of its predisposing causes as sinusitis and otitis media. Small abscess less than 2cm can be treated with antibiotics. Complete evacuation of pus and excision of abscess wall after performing craniotomy along with appropriate antibiotics is gold standard management of brain abscess in children.

## INTRODUCTION

Brain abscess is defined as focal suppurative process within the brain parenchyma that begins as a localized area of cerebritis and changes into collection of pus surrounded by a well vascularized capsule. Infectious process that affects the CNS may threaten vital neurological functions and even life itself. The prognosis of these patients has improved over the past 25 years in large measure due to technological advances in diagnostic and treatment modalities as new generation of antibiotics, CT and MRI. However, in spite of these advances, CNS infections continue to add to neurological morbidity and mortality. 1

The common predisposing factors are paranasal sinusitis, otitis media, trauma, brain surgery and cyanotic heart diseases. Mode of entry of causative microorganism is contiguous spread from infected paranasal sinuses and otitis media, hematogenous in endocarditis due to cyanotic heart diseases and direct inoculation in brain during trauma and surgery.^2^ Fever, headache, vomiting, seizures are common presentations. Altered level of consciousness, neck rigidity and focal neurological deficits may also be present.^3^ Intraventricular rupture of abscess and brain herniation may be fatal complications. An abscess has a stereotyped appearance and ring enhancement on contrast enhanced CT.^4^ Diffusion waited MRI may be helpful in diagnosing brain abscess even at cerebritis stage and differentiating it from other cystic brain lesions.^5^ Small abscess and cerebritis stage respond to parenteral antibiotics and may be treated completely with medical management only without any surgical intervention.^6^ Surgical treatment of brain abscess is aspiration of pus or craniotomy and excision of abscess followed by antibiotic therapy.^7^ Burr hole is made at Kocher’s point for abscess in frontal lobe, Keen’s point for temporoparietal region and at Frasier’s point for occipital region. Craniotomy was performed according to location of abscess.

Choice of surgical procedure depends upon surgeon and is debatable. Aspiration of pus is minimal invasive technique and can be repeated as well, while craniotomy and excision of abscess is an extensive surgical procedure requires long anesthesia.^8^ This study was carried out with an objective to assess the outcome of various surgical options of management of brain abscess.

## METHODS

This is prospective observational study conducted in the Department of Paediatric Neurosurgery at Children’s Hospital and Institute of Child Health Multan for a period of three years from July 2014 to June 2017 after approval from ethical committee dated February 26, 2019. All the patients suffering from brain abscess were admitted throughout patient department or referred from department of paediatric medicine of these hospitals. After taking history and clinical examination, necessary investigations like complete blood cells count and CT scan brain plain and with contrast were performed. MRI was performed if needed. Patients in cerebritis stage of disease or small abscess less than 2cm without causing mass effect were referred to respective department of paediatric medicine for conservative medical management and not included in this study.

Surgical intervention was planned in those patients having brain abscess with size more than 2cm, causing mass effects neurological deficit, at aliquant area or with signs of raised intracranial pressure and these patients were included in the study. Mannitol (20%) was used if signs of raised intracranial pressure were present. Meropenum, vancomycin and metronidazole were started intravenously. Patients were assessed for fitness for general anesthesia and surgical intervention. Pus was aspirated with needle through open anterior fontanelle in infant age group. A burr hole was made at appropriate site to aspirate pus with brain cannula in children above one year of age. Craniotomy for excision of abscess was performed in case of thick loculated septated pus not aspirated through burr hole. In case of multiple abscesses, only larger size abscesses were aspirated. Pus was sent for culture and sensitivity and antibiotics were changed accordingly. Check scan was performed after one week if clinical features of recollection of abscess appeared during the course of therapy. Intravenous antibiotics were continued for 4 to 6 weeks and shifted to oral rout for another. Four to six weeks depending upon clinical and radiological response. Follow-up CT scan was obtained at the completion of therapy routinely and in between if required.

### Statistical analysis

Data was collected on a pre-designed performa and analyzed through statistical package for social sciences (SPSS-20). The results were compared with national and international literature.

## RESULTS

A total of 25 patients suffering from brain abscess were managed. Seventeen (68%) were male and 8(32%) female. Age range was up to 14 years including four infants. Fever and vomiting were present in all 25 (100%) patients, headache in 21 (84%) seizure in 10 (40%) focal neurological deficit in 6 (24%) and irritability in 4 (16%) patients.

**Fig.1:**
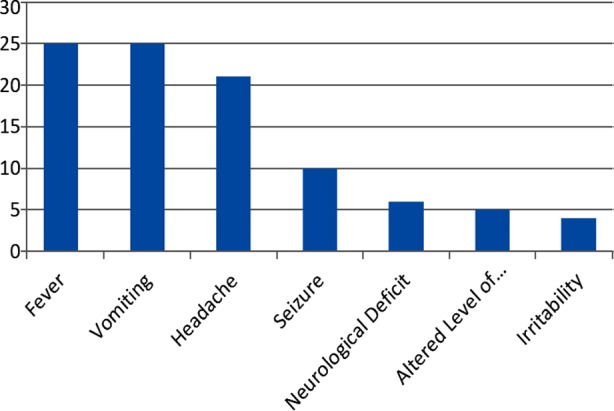
Clinical presentation of Children with Brain Abscess (N = 25).

**Table-I:** Predisposing causative factor.

Causative factor	No. of Patients	Percentage
Paranasal sinusitis	9	36%
Otitis media	7	28%
Endocarditis	4	16%
Brain trauma	2	8%
Unknown	3	12%

Total	25	100%

Paranasal sinusitis was predisposing causative factor in 9(36%), otitis media in 7 (28%), endocarditis secondary to cyanotic heart disease in 4 (16%), brain trauma in 2 (8%) and in 3(12%) patients no causative factor identified.

Abscess was present in frontal lobe in 9 (36%), temporoparietal region in 8 (32%), posterior fossa in 5 (20%) and multiple abscess in 3 (12%).

Pus was aspirated with needle through open anterior fontanelle in 4 (16%) patients less than one year of age, with brain cannula after making a burr hole in 16 (60%) patients and craniotomy performed for excision of thick multiloculated septated pus in 6 (24%) patients. Three (12%) patients expired, two after craniotomy and one after aspiration of abscess.

## DISCUSSION

This study was performed in children age group up to 14 years. A total of 25 patients suffering from brain abscess were managed and included in the study. Twenty-one children were between 1-14 years of age and four were infants under one year of age. Seventeen (68%) were male and 8(32%) female with male to female ratio 2:1. 9. Muzumdar D et al. mentioned that brain abscess can develop at any age, 71% in male and 29% in female with male to female ratio of almost 2:1.^9^ Ranjith et al. mentioned it to be more common in younger age group mostly in first three decades.^7^

Fever and vomiting were present in all 25 (100%) patients, headache in 21 (84%) seizure in 10 (40%), focal neurological deficit in 6 (24%) and altered level of conciseness in 5 (20%). Four patients were infants unable to speak presented with irritability and reluctant to feed. Eva Tonon et al. reported fever in 79%, headache in 69%, neurological deficit in 66%, seizer in 27% and altered level of conciseness in (21%).^10^ Chuang et al. mentioned seizers after bacterial brain abscess and outcome of its management.^11^

**Table-II:** Region of location of brain abscess

Location area	No. of Patients	Percentage
Frontal lobe	9	36%
Temporoparietal	8	32%
Posterior fossa	5	20%
Multiple abscess	3	12%

Total	25	100%

**Table-III:** Treatment modalities.

Treatment modality	No. of Patients	Percentage
Needle aspiration through fontanelle	4	16%
Burr hole aspiration with brain cannula	15	60%
Craniotomy and excision of abscess	6	24%

Paranasal sinusitis was predisposing causative factor in 9(36%), otitis media in 7 (28%), endocarditis secondary to cyanotic heart disease in 4 (16%), brain trauma in 2 (8%) and in 3(12%) no underlying cause was found. Muzumdar D et al. also mentioned paranasal sinusitis as a leading predisposing factor followed by otitis media.^9^ Brain abscess without any known underlying cause has been reported by Stanescu GL et al.^12^ Brain abscess in immune compromised patient was not noted in this study but it is reported in literature by Nelson et al.^13^ Similarly abscess may develop after brain surgery as mentioned by Yang et al.^14^, but not noted in this study. Abscess was present in frontal lobe in 9 (36%), temporoparietal region in 8 (32%), posterior fossa in 5 (20%) and multiple abscess in 3 (12%). Eva Tonon et al. reported location of abscess in temporal lobe 36%, frontal 30%, partial 26%, and occipital 8%.^10^ Ashraf et al. reported location wise chances of brain abscess 46% in frontal, 28% in temporoparietal, 8% in occipital region and 8% at multiple areas.^15^

Culture was positive in 12 (48%) and no growth obtained in 13(52%) cases. Ashraf et al. reported culture positive in 36% and negative in 64%.^15^ Streptococcus milleri was most common organism followed by staphylococcus aureus and *E.coli*. Streptococcus milleri was the commonest organism isolated from pus in other studies conducted by Ashraf et al.^15^ Atiq et al.^16^ and Sineviratne et al.^17^ Staphylococcus aureus was the commonest organism reported by Bhand et al.^18^ Muzumdar D et al. reported bacteroides as common microorganism followed by peptostreptococcus and streptococcus.^9^ Tonon et al. reported streptococci viridians followed by staphylococcus aureus, gram-negative bacilli, and anaerobes as common causative microorganism.^10^

Pus was aspirated by an appropriate size needle in 4 (16%) patients within one year of age having open anterior fontanelle and pus located in frontal and temporoparietal region. Burr hole was performed and pus aspirated with brain cannula in 15 (60%) and craniotomy performed for excision of thick pus in 6 (24%). Same surgical intervention protocol is mentioned by Brouwer MC et al.^4^ Ranjith et al.^7^ and Muzumdar D et al.^9^ Eva Tonon et al. mentioned craniotomy for brain abscess as obsolete procedure.^10^

Three (12%) patients expired, two after craniotomy and one after aspiration of abscess. With improvement in diagnostic technique new antibiotic and surgical techniques outcome of management of disease has improved and mortality has been reduced.^7^,^9^ Eva Tonon et al.^10^ mentioned 8% mortality, Prusty^19^ reported it up to 17% and Ashraf et al. found no mortality in early postoperative period after aspiration of brain abscess.^15^ Outcome of management of brain abscess was assessed clinically and with follow up CT scan in every patient and same protocol is mentioned by Nathoo et al.^20^ Follow up ultrasonography through open anterior fontanelle in four infants was performed in this study. It can be performed through burr hole made for initial surgery as reported by Hayashi et al.^21^

### Limitations of the study

It includes a small sample size. Further studies with a larger sample size are suggested.

## CONCLUSIONS

As paranasal sinusitis and chronic otitis media are common in developing countries and these are common predisposing factors of brain abscess so early and complete treatment of these causes may reduce chances of brain abscess to develop. Early diagnosis of suspected cases of brain abscess and prompt empirical antibiotic therapy should be started immediately in every case of brain abscess even in cerebritis stage. Small abscess less than 2cm can be managed without surgical intervention. Minimal invasive surgical procedure of aspiration is effective and relatively safe. Craniotomy and excision of abscess is preferred in case of thick, septated and multiloculated pus.

### Authors’ Contribution:

**IUK**: conceived, designed and did statistical analysis & editing of manuscript and takes the responsibility and is accountable for all aspects of the work in ensuring that questions related to the accuracy or integrity of any part of the work are appropriately investigated and resolved.

**AL**: did data collection and manuscript writing.

**MA:** did data analysis and manuscript writing.

**MKC:** Manuscript writing.

**SS:** Manuscript writing and proof reading.
